# Antigen epitopes of animal coronaviruses: a mini-review

**DOI:** 10.1186/s44149-023-00080-0

**Published:** 2023-05-17

**Authors:** Mingjun Su, Guanghui Zheng, Xiangwen Xu, Houhui Song

**Affiliations:** Key Laboratory of Applied Technology On Green-Eco-Healthy Animal Husbandry of Zhejiang Province, Zhejiang Provincial Engineering Research Center for Animal Health Diagnostics & Advanced Technology, Zhejiang International Science and Technology Cooperation Base for Veterinary Medicine and Health Management, China-Australia Joint Laboratory for Animal Health Big Data Analytics, College of Animal Science and Technology & College of Veterinary Medicine of Zhejiang, A&F University, 666 Wusu Street, Lin’an District, Hangzhou, 311300 Zhejiang Province China

**Keywords:** Animal coronavirus, Antigen epitope, B-cell epitope, T-cell epitope, Immune responses, Vaccines

## Abstract

Coronaviruses are widespread in nature and can infect mammals and poultry, making them a public health concern. Globally, prevention and control of emerging and re-emerging animal coronaviruses is a great challenge. The mechanisms of virus-mediated immune responses have important implications for research on virus prevention and control. The antigenic epitope is a chemical group capable of stimulating the production of antibodies or sensitized lymphocytes, playing an important role in antiviral immune responses. Thus, it can shed light on the development of diagnostic methods and novel vaccines. Here, we have reviewed advances in animal coronavirus antigenic epitope research, aiming to provide a reference for the prevention and control of animal and human coronaviruses.

**Supplementary Information:**

The online version contains supplementary material available at 10.1186/s44149-023-00080-0

## Introduction

Coronaviruses (CoVs) are positive-sense, single-stranded RNA viruses belonging to the *Coronaviridae* family of the Nidovirales order. Based on their genome sequences, they fall into four genera: *alphacoronavirus* (α-CoV), *betacoronavirus* (β-CoV), *gammacoronavirus* (γ-CoV), and *deltacoronavirus* (δ-CoV) (Li 2016; Zhang et al. 2021a). Coronaviruses have a genome size of approximately 25,000–30,000 nucleotides (nt) and consist of at least six open reading frames (ORFs) in the following order: ORF1a, ORF1b, spike (S), envelope (E), membrane (M), and nucleocapsid (N). Some coronaviruses encode hemagglutinin esterase (HE) (Brian and Baric 2005).

**Fig. 1:**
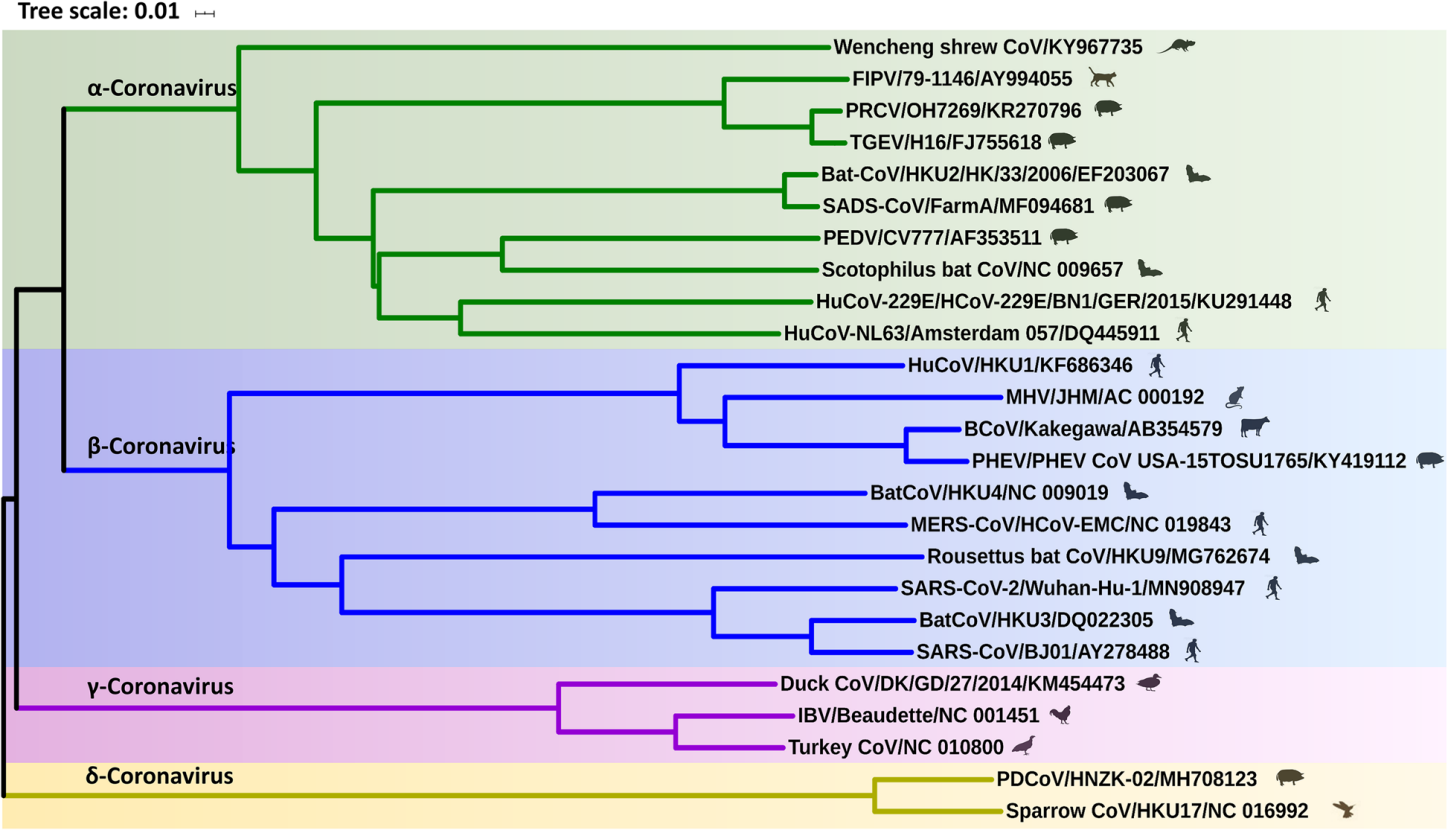
Phylogenetic analysis based on the genome of coronavirus. A neighbor-joining phylogenetic tree was built using the p-distance model and 1,000 bootstrap replicates

Animal coronaviruses (animal CoVs) have a wide range of host tropisms and represent a health risk to livestock production. Since the discovery of infectious bronchitis virus (IBV) in the 1930s, numerous animal CoVs have been identified in pigs, rats, cats, dogs, cattle and horses, belonging to four subgenera of the family *Coronaviridae* (Fig. 1) (Hudson and Beaudette 1932; Woo et al. 2009; Zhang et al. 2021a). It is noteworthy that the prevention and control of emerging and re-emerging CoVs, such as porcine deltacoronavirus (PDCoV), swine acute diarrhea syndrome coronavirus (SADS-CoV), and porcine epidemic diarrhea virus (PEDV), represents a major global challenge (Wang et al. 2019). Antigen epitopes are chemical moieties located on the surface of an antigen molecule that possess a unique structure and antigenic activity. They represent a bioactive region on the antigen molecule that can stimulate the host immune system to produce antibodies or immunogenic lymphocytes and can be recognized by these immune cells. Therefore, the investigation of antigenic epitopes helps our understanding aboutvirus-mediated immune response and provides a basis for the design of antiviral strategies, which is an intense area of virology research. Although the high output of research on animal CoV antigenic epitopes has promoted the study of antigenic epitope diagnostic methods and vaccines, there are still challenges in the identification and application of animal CoV antigen epitopes.

## Distribution of antigen epitopes in animal CoVs

**Fig. 2:**
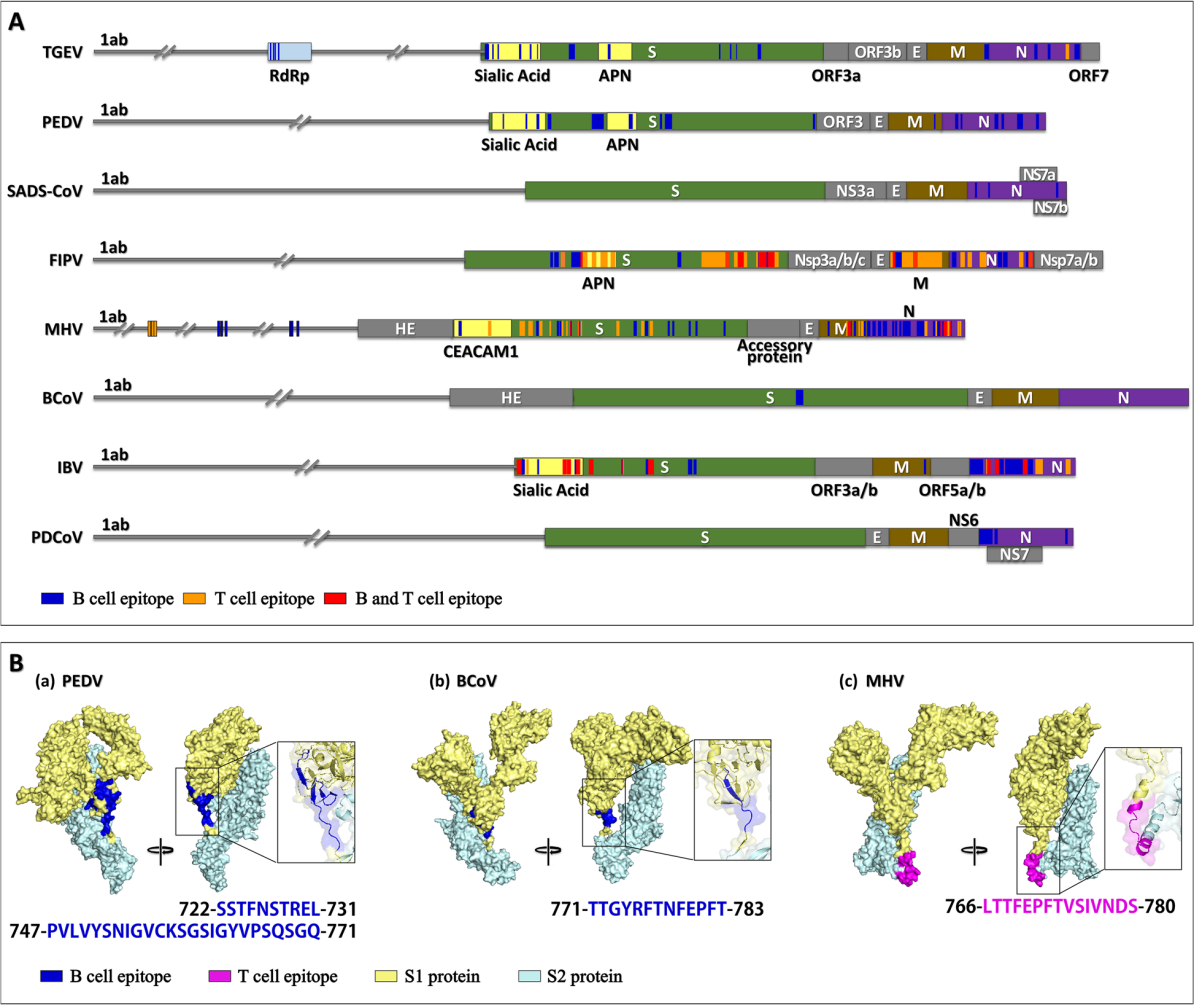
**A** Location of antigen epitopes in animal CoV genomes. **B** Distribution of antigen epitopes in the S1/S2 junction of animal CoV S proteins. The predicted tertiary structures of the S region of PEDV (PDB ID: 6VV5), BCoV (PDB ID: 6NZK), and MHV (PDB ID: 3JCL) were modeled using the open-source modeling server SWISS-MODEL (https://swissmodel.expasy.org/) from the Swiss Institute of Bioinformatics (Biasini et al. 2014). Illustrations of these modeled tertiary structures were obtained using the python-based molecular viewer PyMOL (The PyMOL Molecular Graphics System, V. 1.7.4 Schrödinger, LLC)

Based on the interaction with antigen receptors on cells, epitopes are classified as B-cell or T-cell epitopes, which can induce humoral or specific cellular immunity, respectively. Continuous antigenic epitopes, also known as linear epitopes, are characterized by a continuous stretch of amino acids in the peptide chain. They are typically found on T-cell epitopes and some B-cell epitopes. Conformational antigenic epitopes, on the other hand, are formed by amino acids that are adjacent in space but not continuous in sequence and are only present on B-cell epitopes (Barlow et al. 1986). According to the immune epitope database (IEDB; www.iedb.org) (Vita et al. 2010), multiple B- and T-cell epitopes have been identified in several animal CoVs, including TGEV, PEDV, SADS-CoV, FIPV, MHV, BCoV, IBV and PDCoV (Fig. 2A) (Table S1).

B-cell epitopes have been reported in all eight animal CoVs mentioned above. The main epitopes are on S, M and N proteins (Fig. 2A, Blue), and two B-cell epitopes have been found on NS7a protein of SADS-CoV (Qin et al. 2022). Additionally, four and six B-cell epitopes have been reported on the RNA-dependent RNA polymerase (RdRp) of TGEV and on the RNA polymerase of MHV, respectively (Mathieu et al. 2004; Nogales et al. 2011). Conformational epitopes are epitopes that bind specifically to antibodies that block the cellular receptors used by viruses to bind to cells. This is a critical step in the development of diagnostic reagents. However, only one potential epitope has been detected in the PEDV N protein (residues 18–133) (Wang et al. 2016). T-cell epitopes have been reported in four animal CoVs, including FIPV, MHV, IBV and TGEV (Fig. 2A, Orange). Similar to B-cell epitopes, T-cell epitopes are predominantly located on the S protein, followed by N and M proteins, and two T-cell epitopes have been reported on replicase polyprotein 1ab (pp1ab) of MHV (Croxford et al. 2006; Ercolini et al. 2007).

Coronavirus S protein is incorporated into the viral envelope and facilitates viral entry into target cells. In this process, the surface unit S1 binds to a cellular receptor, while the transmembrane unit S2 enables fusion of the viral membrane to the host cell membrane (Hulswit et al. 2016; Millet and Whittaker 2018). Membrane fusion requires S protein cleavage by host cell proteases at the S1/S2 site, resulting in S protein activation (Hoffmann et al. 2020; Hulswit et al. 2016; Millet and Whittaker 2018). The 3D structure model of S protein showed that the antigen epitopes of PEDV (722–731, 747–771), MHV (766–780), and BCoV (711–783) were identified at the S1/S2 junction (Gillam and Zhang 2018; Khanolkar et al. 2010; Kong et al. 2020; Okda et al. 2017; Sun et al. 2008; Vautherot et al. 1992) (Fig. 2B).

## Applications of animal CoV antigen epitopes

### Application of antigen epitopes in vaccines

Antigenic epitopes can induce humoral immunity or specific cellular immunity that is crucial for inducing antiviral immune responses. They are commonly deployed in the development of safe and effective vaccines. Currently, the known animal CoV antigen epitopes offer a basis for the development of epitope vaccines (Table 1).

**Table 1:** Application of animal CoV antigen epitopes

Coronaviruses (protein)	Sequence	Application	References
PEDV (S-COE)	Y^748^SNIGVCK^755^	Vaccine: production of neutralizing antibody (used HBcAg as vector)	Gillam and Zhang 2018
PEDV (S)	T^592^SLLASACTIDLFGYP^607^	Vaccine: production of neutralizing antibody	Sun et al. 2019
FIPV (S)	N^621^NYLTFNKFCLSLSPVGANC^640^	Vaccine: induce Th1 activity	Takano et al. 2014
FIPV (M)	V^81^YGIKMLIMWLLWPIVLALT^100^	Vaccine: induce Th1 activity	Takano et al. 2014
FIPV (N)	G^81^QRKELPERWFFYFLGTGPH^100^	Vaccine: induce Th1 activity	Satoh et al. 2011
IBV (S)	S^413^RIQTATDP^421^, ^517^RNATGSQP^525^, G^45^AYAVVNV^52^, S^413^RIQTATQP^421^	Vaccine: stimulate CD8^ +^ T-cell proliferation and IFN-γ secretion	Tan et al. 2016
TGEV (S)	TGEV S-A site (533–705)	Vaccine: increase the Th1 and Th2 cytokine levels	Gebauer et al. 1991
MHV (S: S1/S2 junction)	L^766^TTFEPFTVSIVNDS^780^	Vaccine: induces CD4 T-cell response in mice	Khanolkar et al. 2010
PEDV (S-COE)	T^592^SLLASACTIDLFGYP^607^	Diagnosis: mABs 4D8F10 and 6F3E3 recognize the COE, and highly conserved	Sun et al. 2019
TGEV (S)	T^592^SLLASACTIDLFGYP^607^	Diagnosis: highly conserved	Gebauer et al. 1991
MHV (N)	I^24^LKKTTWADQTERGL^38^ R^357^FDSTLPGFETIMKVL^372^	Diagnosis (ELISA): more sensitive than the commercial tests	Asano et al. 2011
IBV (S)	TGEV (S1: 166–247, S1: 501–515, S2: 8–30)	Diagnosis (ELISA): more sensitive and specific than the commercial tests	Ding et al. 2015

 S proteins, especially the S1 of PEDV, are a key target for virus neutralization and vaccine development (Hain et al. 2016; Makadiya et al. 2016; Oh et al. 2014; Subramaniam et al. 2017). The B-cell epitope Y^748^SNIGVCK^755^ on S1 protein of PEDV has been validated for potential use in vaccine development (Gillam et al. 2018; Okda et al. 2017). Gillam reported that a Y^748^SNIGVCK^755^-based epitope vaccine that used hepatitis B virus core antigen (HBcAg) as a vector efficiently elicited the production of anti-PEDV neutralizing antibodies in mice (Gillam and Zhang 2018). PEDV collagenase equivalent domain (COE), a crucial antigenic region within the viral S protein, has been widely used in the development of subunit vaccines (Ge et al. 2012; Ma et al. 2018). Sun reported a B-cell epitope, T^592^SLLASACTIDLFGYP^607^, within the COE that was conserved among G1 and G2 PEDV strains and may mediate the production of neutralizing antibodies (Sun et al. 2019).

Developing an FIP-preventive vaccine requires an antigen that does not induce antibody-dependent enhancement (ADE), and T helper (Th) 1 activity plays an important role in protecting against FIPV infection (Gelain et al. 2006; Kiss et al. 2004; Pedersen 2009; Weiss and Cox 1989). Satoh and Takano identified Th1 and linear immunodominant antibody-binding epitopes in the S1 domain, M protein and N protein of FIPV (Satoh et al. 2011; Takano et al. 2014). It has been shown that the T-cell epitopes N^621^NYLTFNKFCLSLSPVGANC^640^ (II-S1-24), V^81^YGIKMLIMWLLWPIVLALT^100^ (I-M-9), and G^81^QRKELPERWFFYFLGTGPH^100^ (NP-7) strongly induce Th1 activity. This knowledge may guide the development of epitope vaccines against FIPV infection.

The broad cytotoxic T lymphocyte (CTL) response against IBV is a crucial factor in viral replication control (Cavanagh 2007; Collisson et al. 2000). Tan reported that four CTL epitopes of IBV (S^413^RIQTATDP^421^, S^517^RNATGSQP^525^, G^45^AYAVVNV^52^ and S^413^RIQTATQP^421^) can stimulate CD8^+^ T-cell proliferation and IFN-γ secretion (Tan et al. 2016). In vivo studies revealed that a poly-CTL epitope-based vaccine (pV-S1T) constructed by inserting the nucleotide sequences of the above four CTL epitopes into the pVAX1 vector provided 90% protection against an avirulent IBV strain.

The N-terminus of TGEV S protein contains four antigenic sites, A, B, C and D, which are involved in the stimulation of neutralizing antibodies (Delmas et al. 1990). Past studies have shown that the A site is predominantly responsible for stimulating neutralizing antibodies (Correa et al. 1990; Delmas et al. 1990; Laviada et al. 1990; Meng et al. 2013, 2011; Zhao et al. 2013). Yuan constructed a recombinant swinepox virus (rSPV-SA) expressing the TGEV S-A site (533–705) (Yuan et al. 2015). The results from passive immunity protection test of newborn piglets revealed that the recombinant live-vector vaccine rSPV-SA 100% protected piglets from SPV infection, and no significant clinical symptoms were observed in the rSPV-SA treatment group during this experiment. The antigen epitope M^537^KSGYGQPIA^547^, which is located in the TGEV S-A site, has been identified as a B-cell epitope of TGEV (Gebauer et al. 1991). However, whether this epitope can produce neutralizing antibodies remains unclear.

Antigen epitopes on the S1/S2 junction of animal CoVs have been shown to stimulate neutralizing antibody production. Okda found that the S1/S2 junction of PEDV is an immunodominant region of S protein with strong neutralizing activity (Okda et al. 2017; 2007). Previous studies have shown that the B-cell epitope Y^748^SNIGVCK^755^ on the S1/S2 junction of PEDV has potential use in vaccine development (Gillam and Zhang 2018). The T-cell epitope L^766^TTFEPFTVSIVNDS^780^ on the S1/S2 junction of MHV efficiently induces CD4 T-cell response in mice (Khanolkar et al. 2010). Vautherot found that the B-cell epitope T^771^TGYRFTNFEPFT^783^, which is on the S1/S2 junction of BCoV, is a potential immunodominant region (Vautherot et al. 1992). The above studies suggest that these antigen epitopes located on the S1/S2 junction might be used for developing epitope vaccines.

### Application of antigen epitopes in diagnosis

Approaches based on antigen epitopes, which permit a high epitope density and careful choice of unique specific epitopes, have been used in the detection of antibodies against viruses and have achieved both high sensitivity and greater specificity in results (Anandarao et al. 2006; Gómara et al. 2010; He et al. 2011). Antigen epitope-based diagnosis relies on two strategies: one uses a highly conserved dominant antigen epitope, and the other combines multiple epitopes (Table 1).

The COE epitope T^592^SLLASACTIDLFGYP^607^, belonging to the B-cell epitope of PEDV, is highly conserved between G1 and G2 PEDV strains. The mAbs 4D8F10 and 6F3E3 that detect the COE epitope are suitable for PEDV by binding to the conserved epitope (Sun et al. 2019). Similarly, the TGEV B-cell epitope T^592^SLLASACTIDLFGYP^607^, which is highly conserved in coronaviruses, can be used as an antigenic peptide for diagnosis (Gebauer et al. 1991). Studies on other coronaviruses indicate that the N protein is highly conserved in different strains. Thus, it is widely used as a diagnostic antigen for the development of serologic diagnostic tools (Abdelwahab et al. 2015; Hou et al. 2007; Pradhan et al. 2014; Su et al. 2016). Asano established two indirect enzyme-linked immunosorbent assays (ELISAs) based on the B-cell epitopes I^24^LKKTTWADQTERGL^38^ and R^357^FDSTLPGFETIMKVL^372^, which are located in MHV N protein. ELISAs that rely on these peptides are more sensitive than the commercial tests used to screen laboratory mouse serum for unintended MHV infection (Asano et al. 2011). Diagnostic methods based on multiple antigens or synthetic peptides exhibit improved sensitivity and specificity (Chimeno Zoth and Taboga 2006; Hadifar et al. 2014; Shehata et al. 2012; Shenyang et al. 2011). Ding developed a multiepitope antigen of S protein (166–247 aa, S1 gene; 501–515 aa, S1 gene; 8–30 aa, S2 gene) for use in a highly sensitive and specific ELISA for detecting IBV-specific antibodies in chicken serum samples (Ding et al. 2015).

## Prospect

Studies on the antigen epitopes on animal CoV have a high research output. However, they are mainly focused on IBV, FIPV, MHV, TGEV and PEDV. The relatively less harmful or newly emerged BCoV, PDCoV and SADS-CoV have not been studied extensively. Currently, technology for identification B-cell epitopes is well established. However, it is difficult to obtain high-quality monoclonal antibodies, especially those with neutralizing activity that are capable of recognizing conformational epitopes. For conformational B-cell antigen epitope identification, monoclonal antibodies recognized by whole viruses are more efficient than monoclonal antibodies from recombinant proteins. Identification of T-cell epitopes is crucial in the investigation of cellular immune mechanisms and the development of subunit peptide vaccines. Except for FIPV, MHV and IBV, few studies on T-cell antigenic epitopes of other animal CoVs have been reported. Since T cells only recognize antigenic polypeptides presented by major histocompatibility complex (MHC) molecules on the surface of antigen presenting cells (APCs), identification of T-cell epitopes is more challenging. Given that some coronaviruses, such as FIPV, have ADE characteristics, immune responses based on humoral immunity may not be suitable for preventing animal CoVs. Thus, further research on animal CoV T-cell epitopes is needed.

Relative to traditional vaccines, epitope vaccines are safer, nontoxic and stable and can more directly elicit immune responses against pathogenic microorganisms. However, in coronaviruses, applications of antigenic epitopes are poorly studied. Although several B- and T-cell epitopes that can induce antiviral immune responses have been identified, studies on the application of antigenic epitope-based diagnosis and vaccines are inadequate, with most having been done in vitro or in nonhost animals without evaluation of immune response and protection in susceptible animal hosts. The identification of different antigenic epitopes on various strains of the same virus limits the application of antigenic epitopes. Generally, antigenic epitopes are located on the surface of viral proteins with hydrophilicity and surface accessibility and are prone to certain mutations due to frequent contact with the external environment. In particular, coronavirus S proteins are located in the outermost layer of the virus and are most susceptible to mutation, resulting in poor conservation of S protein antigenic epitope. For example, Zhang identified that the residues of S protein at position 55–64 were specific for the recognition of PEDV classical G1 strains, whereas the residues at position 157–164 showed specificity to PEDV emerging G2 strains (Zhang et al. 2023). Thus, it is crucial to identify the conserved antigen epitopes, especially neutralizing epitopes, between different strains of the same coronaviruses.

Epitope vaccines based on multiepitope peptides can overcome the problem of low conservation between epitopes from different strains and trigger stronger immune responses. However, for animal CoV, the development of diagnoses and vaccines based on multiple epitopes is inadequate. Since multiepitope vaccines are based on different viral epitopes in tandem, they are suited for the development of universal vaccines against multiple animal CoVs that infect the same host, such as TGEV, PEDV, SADS-CoV and PDCoV. For example, multiple studies have shown that some people who have not been exposed to severe acute respiratory syndrome coronavirus 2 (SARS-CoV-2) have preexisting reactivity to SARS-CoV-2 sequences and that preexisting reactivity to SARS-CoV-2 is mediated by memory T cells (Braun et al. 2020; Grifoni et al. 2020; Le Bert et al. 2020). Cross-reactive T cells have been shown to specifically recognize a SARS-CoV-2 epitope as well as the homologous epitope from a common cold coronavirus (e.g., human coronavirus-OC43/229E/HL63/HKU1) (Mateus et al. 2020). Additionally, epitope vaccines based on B- and T-cell epitopes can elicit humoral and cellular immune responses, resulting in a stronger antiviral immune response, which deserves intensive investigation.

During the virus‒host game, the host produces neutralizing antibodies to defend against viral invasion by recognizing viral antigenic epitopes. Meanwhile, the virus achieves immune escape by continuously mutating to reduce the neutralizing ability of host antibodies. When a virus enters a cell, the host immune system triggers the production of neutralizing antibodies that can effectively block the virus's ability to interact with susceptible cell receptors, interfere with the fusion of the virus with the cell membrane, and form immune complexes that are efficiently cleared by the immune system (Murin et al. 2019). S protein of SARS-CoV-2 mediates virus entry into cells and is the main recognition target of neutralizing antibodies. Mutations in individual amino acid sites on S protein can cause coronavirus to escape neutralizing antibodies. For example, the delta variant of SARS-CoV-2 has mutations in S protein that result in a sixfold reduction in the neutralizing ability of serum neutralizing antibodies compared to wild-type SARS-CoV-2 (Zhang et al. 2021b). Additionally, the clinically approved monoclonal antibodies bamlavinimab and imdevimab for the treatment of SARS-CoV-2-associated disease have shown a 1,000-fold and 50-fold reduction in neutralizing ability against the delta variant, respectively (Mlcochova et al. 2021). Therefore, identifying key antigenic epitope conserved regions of coronaviruses, such as the conserved receptor binding domain and the S1/S2 junction conserved region, is crucial for the development of broad-spectrum neutralizing antibodies against coronaviruses.

## Supplementary Information

**Additional file 1: Table S1.** IEDB inventory of B-cell and T-cell epitopes.

## Data Availability

Not applicable.
